# Supplementary material for: MiR-146a-5p Expression in Peripheral CD14+ Monocytes from Patients with Psoriatic Arthritis Induces Osteoclast Activation, Bone Resorption, and Correlates with Clinical Response
Source: J Clin Med. 2019 Jan 17;8(1):110. doi: 10.3390/jcm8010110 (PMC6352034; doi:10.3390/jcm8010110)
Supplement: Supplementary file 1 [file jcm-08-00110-s001.pdf]

Table S1. qPCR validation result.

Sample	Delta *Ct* (Δ*Ct*)
NC 1	5.33
NC 2	7.22
NC 3	7.27
NC 4	7.79
NC 5	7.71
NC 6	7.75
NC 7	8.61
NC 8	7.74
NC 9	8.38
NC 10	8.75
NC 11	10.06
NC 12	9.05
NC 13	8.8
NC 14	7.78
NC 15	7.88
NC 16	8.35
NC 17	7.6
NC 18	9.04
NC 19	9.09
NC 20	7.32
NC 21	9.48
NC 22	8.09
NC 23	6.89
NC 24	5.48
NC 25	6.16
NC 26	8.07
NC 27	6.49
NC 28	5.59
NC 29	7.48
NC 30	7.28
NC 31	5.23
NC 32	5.56
NC 33	7.52
NC 34	7.77
PsA 1	3.8
PsA 2	4.71
PsA 3	5.54
PsA 4	4.32
PsA 5	5.22
PsA 6	5.89
PsA 7	6.72
PsA 8	4.97
PsA 9	5.21
PsA 10	7.62
PsA 11	5.56
PsA 12	7.54
PsA 13	6.86
PsA 14	6.27
PsA 15	6.55
PsA 16	6.07
PsA 17	6.22
PsA 18	6.34
PsA 19	6.94

PsA 20	6.54
PsA 21	7.24
PsA 22	8.9
PsA 23	8.4
PsA 24	6.71
PsA 25	8.64
PsA 26	7.1
PsA 27	8.97
PsA 28	7.3
PsA 29	7.66
PsA 30	8.34
PsA 31	6.92
PsA 32	6.14
PsA 33	6.5
PsA 34	7.57

We used qPCR assay to validate the expression level of miR-146a-5p in all samples. The Delta Ct values were standardized with the Ct values of internal Normal U6.
